# Causal inference and effect estimation using observational data

**DOI:** 10.1136/jech-2022-219267

**Published:** 2022-09-06

**Authors:** Erik Igelström, Peter Craig, Jim Lewsey, John Lynch, Anna Pearce, Srinivasa Vittal Katikireddi

**Affiliations:** 1 MRC/CSO Social and Public Health Sciences Unit, University of Glasgow, Glasgow, UK; 2 Health Economics and Health Technology Assessment, School of Health and Wellbeing, University of Glasgow, Glasgow, UK; 3 School of Public Health, The University of Adelaide, Adelaide, South Australia, Australia

**Keywords:** methods, research design, study design, epidemiology, statistics

## Abstract

Observational studies aiming to estimate causal effects often rely on conceptual frameworks that are unfamiliar to many researchers and practitioners. We provide a clear, structured overview of key concepts and terms, intended as a starting point for readers unfamiliar with the causal inference literature. First, we introduce theoretical frameworks underlying causal effect estimation methods: the counterfactual theory of causation, the potential outcomes framework, structural equations and directed acyclic graphs. Second, we define the most common causal effect estimands, and the issues of effect measure modification, interaction and mediation (direct and indirect effects). Third, we define the assumptions required to estimate causal effects: exchangeability, positivity, consistency and non-interference. Fourth, we define and explain biases that arise when attempting to estimate causal effects, including confounding, collider bias, selection bias and measurement bias. Finally, we describe common methods and study designs for causal effect estimation, including covariate adjustment, G-methods and natural experiment methods.

## Introduction

There are three core tasks of epidemiology—to describe health states, predict outcomes and identify causes.^1 2^ Methodological developments to estimate causal effects using observational data have drawn on diverse disciplines, including epidemiology, statistics, econometrics and computer science, with varied terminology used.^3–5^ Previous glossaries in this series^6 7^ have dealt with the process of assessing causality across a body of evidence, which has long been an essential part of epidemiological research.^8^ However, past glossaries have not covered many concepts underlying more recent methods for causal effect estimation based on counterfactual theory and the potential outcomes framework. Understanding these concepts is important for those engaging with and conducting epidemiological and public health research. Although we focus on observational study designs, the same principles and issues are also applicable to ‘non-ideal’ randomised controlled trials (RCTs), for example with attrition or imperfect adherence, when estimating anything other than an intention-to-treat effect.

## Key concepts and frameworks

### Counterfactual theory of causation

A *counterfactual* is a ‘what-if’ statement that describes what would have been the case under different circumstances than those observed—hence ‘counter to the facts’. According to a *counterfactual theory of causation*, causal claims (using words like ‘cause’ or ‘prevent’) can be expressed in counterfactuals. For example, ‘Bringing an umbrella prevented me from getting wet’ could be rephrased either as ‘If I had not brought an umbrella, I would have got wet’ (using a *deterministic* interpretation of causation, where not bringing an umbrella always leads to getting wet) or ‘If I had not brought an umbrella, I would have been more likely to get wet’ (using a *probabilistic* interpretation of causation, where not bringing an umbrella leads to a higher likelihood of getting wet).^9^ The second, probabilistic interpretation is the most relevant and widely used in epidemiology.

### Potential outcomes

The *potential outcomes framework* (Rubin or Neyman-Rubin causal model) uses mathematical notation to describe counterfactual outcomes and can be used to describe the causal effect of an exposure on an outcome in statistical terms.^10^ The terms *exposure* and *outcome* refer to the central variables of interest where the exposure is thought to have a causal effect on the outcome, which the study seeks to estimate. The exposure may be a treatment, intervention or some other variable that could have taken one of several counterfactual values. In this glossary, an exposure is denoted by ‘A’ (lower case ‘a’ for a particular exposure value) and an outcome by ‘Y’.

If we label an individual’s exposure status as 1 or 0, then $Y^{a=1}$ denotes the *potential outcome* if they had been exposed, and $Y^{a=0}$ denotes the potential outcome if they had been unexposed—this is one of several forms of notation commonly used in the literature, and others are shown in online supplemental table 1. Potential outcomes refer to all possible outcomes that an individual could experience—both those which are observed (factual) and those which are not (counterfactual). Given a binary exposure and a binary outcome, the possible combinations of actual and counterfactual outcomes give rise to four *causal types* 11:

10.1136/jech-2022-219267.supp1 Supplementary data

- ‘Doomed’: would have experienced the outcome regardless of exposure.
- ‘Causative’: would have experienced the outcome if exposed, otherwise not.
- ‘Preventative’: would have experienced the outcome if unexposed, otherwise not.
- ‘Immune’: would not have experienced the outcome regardless of exposure status.

The counterfactual outcomes of a specific individual can never be known, since we can never observe the same individual both exposed and unexposed under the same circumstances (eg, both taking and not taking an umbrella on the same occasion). Instead, we estimate outcomes of groups of people in probabilistic terms, such as the *expected value* (mean) of a continuous outcome:

E(Y)

or the *probability* of a binary outcome:

P(Y=1)

A *conditional expectation* such as E(Y|C=1) denotes the expected value of Y, *given that* another variable C is 1. More generally, an expression such as E(Y|C) can be read as ‘the expected value of Y *conditional on C*’ (ie, ‘holding C constant’ or ‘within levels of C’). *Conditioning on* a variable is analogous to controlling for, adjusting for or stratifying by it (although in practice, different methods of conditioning may have different effects on the results and their interpretation).

### Causal diagrams (directed acyclic graphs)

**Figure 1:**
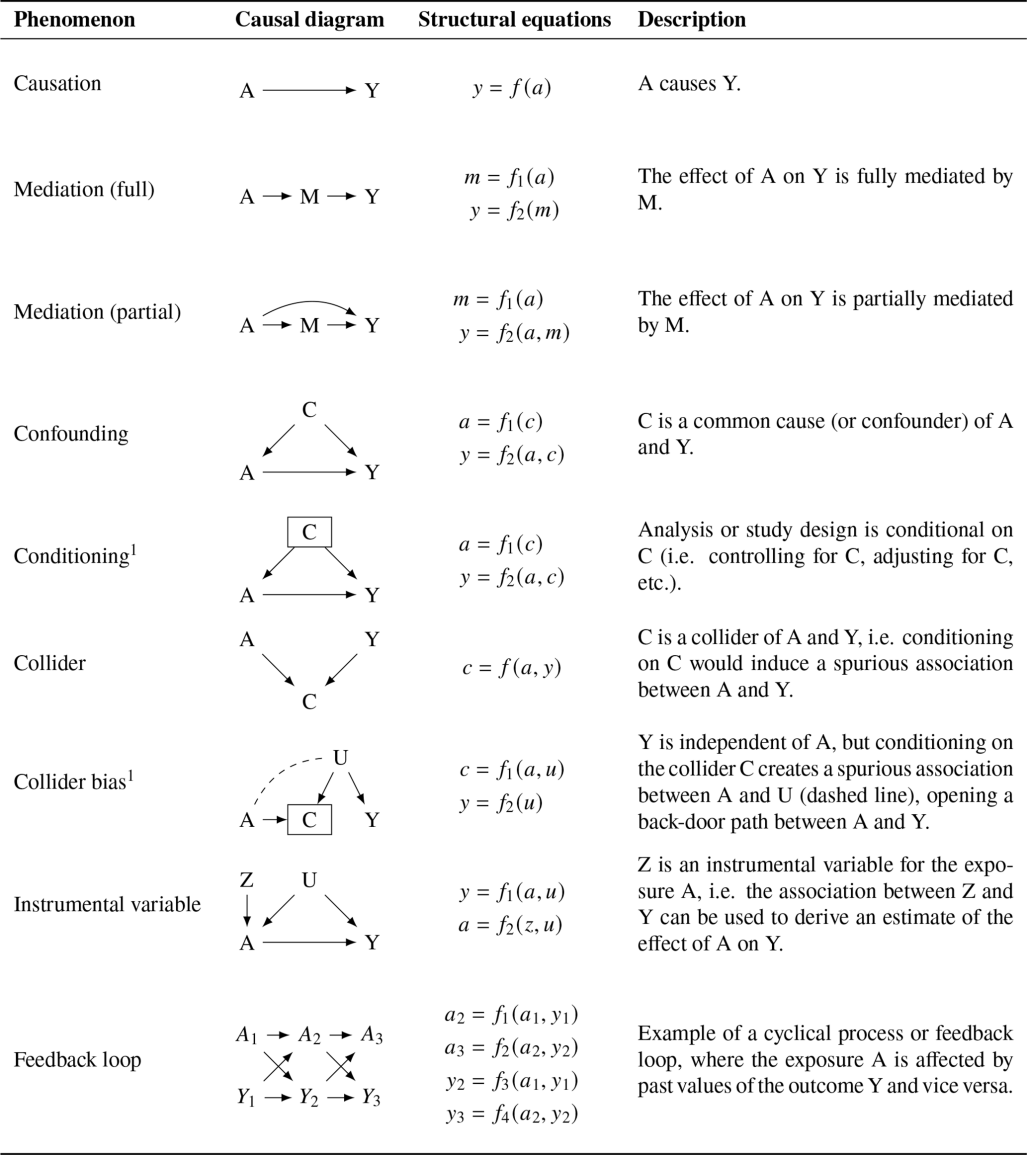
Causal diagrams and equivalent structural equations for common causal phenomena. ^1^Note that conditioning on the variable C is only represented in the causal diagram, not in the structural equations. A: exposure; C: confounder or collider; M: mediator; U: unmeasured confounder; Y: outcome; Z: instrumental variable.

Causal relationships between variables of interest can be described using *causal diagrams* (figure 1).^3 12^ Each *node* represents a variable at a specific point in time, and an *arrow* (sometimes ‘edge’ or ‘arc’) from A to B indicates that A has a causal effect on B; that is, if A had been different, then the expected value or probability of B would have been different. A box drawn around a variable indicates that the study design or analysis conditions on that variable. *Directed acyclic graphs* (DAGs) are causal diagrams where no instantaneous cyclical relationships exist. Causal DAGs (henceforth DAGs) can also be used to represent cyclical processes or feedback loops, using multiple nodes to represent the same variable at different points in time, and this allows cyclical processes or feedback loops to be modelled explicitly (see figure 1).

DAGs represent theories about causal mechanisms underlying a specific research question. The same research question could be represented by multiple DAGs, depending on the assumptions made by the researchers. Relationships between variables in a DAG can also be described using *structural equations*, so called because they describe causal relationships rather than observed associations (figure 1).^13^ A set of structural equations can sometimes be rewritten as a single *reduced form* equation.

## Defining causal effects

The size of a causal effect is the difference in the potential outcomes for a particular population given different counterfactual scenarios (eg, one where everyone is exposed vs one where everyone is unexposed). As with potential outcomes, causal effects cannot be observed at an individual level, so we rely instead on estimating average effects in groups of people. The outcome may be the mean of a continuous variable or the risk of a binary outcome. The *scale* of an effect measure can be either *additive* or *multiplicative* (table 1). For the remainder of this glossary, examples will be given in terms of a binary exposure and a continuous outcome on an additive scale, but the principles apply more generally.

**Table 1:** Potential outcome notation for additive and multiplicative causal effect measures for continuous and binary outcomes

Type of outcome	Scale	Potential outcome notation	Effect measure	Example interpretation
Continuous	Additive	$E\left(Y^{a=1}-Y^{a=0}\right)$	Causal mean difference	‘An average increase in systolic blood pressure by 10 mm Hg’
	Multiplicative	$\frac{E\left(Y^{a=1}\right)}{E\left(Y^{a=0}\right)}$	Causal mean ratio	‘An average increase in systolic blood pressure by a factor of 1.1’ or ‘by 10%’
Binary	Additive	$P\left(Y^{a=1}=1\right)-P\left(Y^{a=0}=1\right)$	Causal risk difference	‘An average increase in the risk of stroke by 5 percentage points’
	Multiplicative	$\frac{P\left(Y^{a=1}=1\right)}{P\left(Y^{a=0}=1\right)}$	Causal risk ratio	‘An average increase in the risk of stroke by a factor of 1.5’

Several causal *treatment effects* can be distinguished, depending on how the exposure is defined and what population is considered (table 2). It is crucial to specify which treatment effect a given study is seeking to estimate (its causal *estimand*), since these can differ substantially in terms of effect size, risk of bias and interpretation.^14^ Deciding which treatment effect is most relevant to the research question and target population is often not straightforward.^4 5^ One way to help clarify what causal effect a study is estimating is to specify a *target trial*, that is, a hypothetical RCT that the study is attempting to emulate.^15 16^

**Table 2:** Definitions of different types of treatment effect

Effect	Potential outcome notation	Description
Average treatment effect (ATE)	$E\left(Y^{a=1}-Y^{a=0}\right)$	The difference between the average outcome when everyone is exposed, and the average outcome when nobody is.
Average treatment effect in the treated (ATT)	$E\left(Y^{a=1}-Y^{a=0}|A=1\right)$	The ATE in the subpopulation of individuals who were actually exposed.
Average treatment effect in the untreated (ATU/ATUT)	$E\left(Y^{a=1}-Y^{a=0}|A=0\right)$	The ATE in the subpopulation of individuals who were actually unexposed.
Intention-to-treat effect (ITT)	$E\left(Y^{z=1}-Y^{z=0}\right)$	Average effect of being assigned to (but not necessarily receiving) the exposure.
Complier average causal effect (CACE) or local average treatment effect (LATE)	$E\left(Y^{a=1}-Y^{a=0}|\begin{array}{c} A^{z=0}=0,\\ A^{z=1}=1 \end{array}\right)$	The ATE among the ‘compliers’, that is, the subpopulation whose exposure status was affected by the assignment mechanism.

*A* denotes actual exposure status (a=1 for exposed, a=0 for unexposed). *Z* denotes assignment to the exposure, which may or may not have been adhered to (*z*=1 for assignment to the exposure, *z*=0 for assignment away from the exposure).

### Effect measure modification

**Figure 2:**
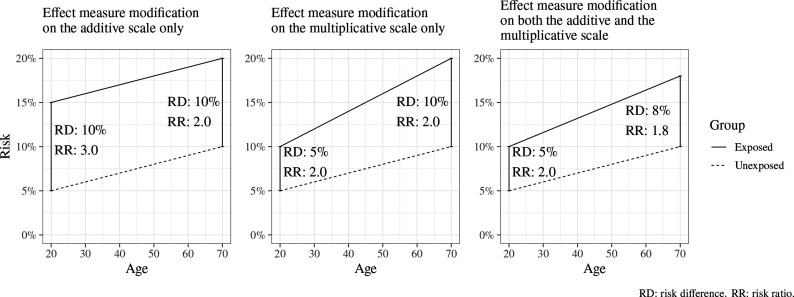
Illustration of effect measure modification by age of the risk of an unspecified outcome, when measured using an additive scale (left), a multiplicative scale (middle) and both additive and multiplicative scales (right). RD: risk difference. RR: risk ratio.

The size of an effect may differ across levels of another variable (eg, gender or age); this is called *effect measure modification* (EMM), and such a variable is an *effect modifier* (or *moderator*).^17 18^ The presence and extent of EMM mathematically depends on the choice of an additive or multiplicative scale linking exposure and outcome; EMM may be present on either one of these scales or both (figure 2). If both the exposure and effect modifier are causes of the outcome, then EMM will always be present on at least one scale.

*Interaction* denotes that the joint effect of two exposures is different from the sum of the individual effects of each exposure. Like EMM, the presence and extent of interaction depends on the choice of an additive or multiplicative scale and does not necessarily have a meaningful causal interpretation. ‘Interaction’ is sometimes used interchangeably with EMM, but it is helpful to think of these as different concepts. Interaction focuses on the joint causal effect of two exposures (eg, the combined effect of smoking and asbestos exposure on lung cancer),^17 19^ while EMM focuses on the effect of one exposure whose effect differs across levels of another variable (eg, the effect of asbestos exposure on lung cancer in smokers vs non-smokers); with EMM, the causal effect of the effect modifier itself is not of interest.

### Mediation

A *mediator* is a variable on the causal pathway between an exposure A and an outcome Y, that is, where A causes the mediator and the mediator in turn causes Y.^17^ *Mediation analysis* aims to quantify how much of the total effect of A on Y is explained by a particular mediator (the *indirect effect*), and how much is not (the *direct effect*).^20 21^ The *controlled direct effect* (CDE) is the effect of the exposure conditional on the mediator, that is, after eliminating any variation in the value of the mediator. Assuming no interaction between exposure and mediator, and no confounding between mediator and outcome, the indirect effect can be obtained by subtracting the CDE from the total effect.^20^

When interaction is present between exposure and mediator, the CDE will take on different values for different levels of the mediator, and the effect obtained by subtracting the CDE from the total effect no longer has a meaningful causal interpretation.^20^ To address this problem, alternative definitions of causal direct and indirect effects have been proposed (see table 3), such that their sum adds up to the total effect even in the presence of interactions, generally by allowing one or more of these effects to include the interaction effect.^20 22 23^ These effect estimands can be defined theoretically in counterfactual terms, but can only be estimated given additional assumptions that are difficult to verify and may lack applicability for estimating policy-relevant mediation quantities (eg, how much the effect of A on Y could be reduced by intervening on the mediator).^24^

**Table 3:** Different types of direct and indirect effects defined using potential outcome notation

Effect	Potential outcome notation	Description
Controlled direct effect	$E\left(Y^{a=1}-Y^{a=0}|M=m\right)$	Effect of changing the exposure, with the mediator fixed at a specific level (*m*).
Natural direct effect^22^ Pure direct effect^37^	$E\left(Y^{a=1,M^{a=0}}-Y^{a=0,M^{a=0}}\right)$	Effect of changing the exposure, with the mediator fixed at whatever (counterfactual) value it would have if the exposure were absent.*
Natural indirect effect^22^ Total indirect effect^37^	$E\left(Y^{a=1,M^{a=1}}-Y^{a=1,M^{a=0}}\right)$	Effect of changing the mediator between the values it would have with and without the exposure, with the exposure status fixed at exposed.*
Pure indirect effect^37^	$E\left(Y^{a=0,M^{a=1}}-Y^{a=0,M^{a=0}}\right)$	Effect of changing the mediator between the values it would have with and without the exposure, with the exposure status fixed at unexposed.*
Total direct effect^37^	$E\left(Y^{a=1,M^{a=1}}-Y^{a=0,M^{a=1}}\right)$	Effect of changing the exposure, with the mediator fixed at whatever (counterfactual) value it would have if the exposure were present.*^24^

*Requires ‘cross-world’ independence assumption. See Ref. 24.

## Identifying causal effects

### Identifying assumptions

Causal effects are impossible to measure directly, since they involve comparing unobserved counterfactual outcomes that would have happened under different circumstances. A causal effect is *identifiable* if it can be estimated using observable data, given certain assumptions about the data and the underlying causal relationships. Such *identifying assumptions* typically cannot be fully tested statistically but have to be justified based on theory and/or existing evidence about the real-world processes under study.

The *exchangeability* (or ‘no confounding’) assumption requires that individuals who were exposed and unexposed have the same potential outcomes on average.^25^ This allows the observed outcomes in an unexposed group to be used as a proxy for the counterfactual (unobservable) outcomes in an exposed group. RCTs strive to achieve exchangeability by randomly assigning the exposure, while observational studies often rely on achieving *conditional exchangeability* (or ‘no unmeasured confounding’), which means that exchangeability holds after conditioning on some set of variables.

The *positivity* assumption requires that every value of exposure was possible (ie, had a non-zero probability) for each individual at the time that exposure was assigned.^26^ When conditioning on other variables, positivity needs to hold for each combination of covariates. This means that for every combination of covariates, it is possible to be either exposed or unexposed. The combination of covariates where this assumption holds can be called the ‘region of common support’. If some combinations are impossible (eg, if a treatment is never prescribed when a particular contraindication is present), this is considered a *structural positivity violation*. The term *random positivity violation* is used when a combination is possible, but missing from the study sample by chance. The term ‘positivity’ may refer to both of these or only to structural positivity; the latter is usually more relevant in theoretical causal inference literature.

The *consistency* assumption (unrelated to Bradford Hill’s ‘consistency’ criterion^8^) requires that the exposure is sufficiently well defined, so that each individual has one potential outcome for each level of the exposure.^27 28^ This assumption (sometimes called ‘treatment-variation irrelevance*’*) is violated if there are multiple different versions of the exposure (eg, dosages of a drug or reasons for becoming unemployed) with different causal effects. In this case, the estimated effect will be an average of these different causal effects. In practice, perfect consistency is often impossible to achieve, and the crucial question is then whether these differences are small enough for the averaged estimate to be meaningful.

The *non-interference* assumption requires that an individual’s potential outcomes (and hence the causal effect of the exposure for that individual) does not depend on the exposure status of anyone else.^10 29^ This assumption can be violated by ‘spillover effects’ of some exposures (eg, vaccination), where an individual’s outcomes are affected by the exposure status of those around them. The consistency and non-interference assumptions together are sometimes known as the *stable unit treatment value assumption*.

### Threats to causal identification

#### Confounding bias

*Confounding* bias can arise when exposure and outcome share an uncontrolled common cause. In a DAG, confounding arises when variables are connected by a *back-door path*, that is, a path between A and Y that remains even if all arrows pointing away from A are removed. A back-door path can be *blocked* by conditioning on one or more variables along the path (unless they are colliders; see below). Conditioning on every confounding variable on the path is theoretically not necessary as long as the path as a whole is blocked, although mismeasurement of confounding variables may warrant adjustment for multiple variables. In other disciplines, confounding bias is referred to as *omitted variable bias*, *endogeneity* and *selection into treatment*.

*Observed confounders* refer to confounders for which measures are available in the study data. *Residual confounding* is any confounding bias that remains after conditioning on observed confounders, either due to variables not observed in the data (*unmeasured* or *unobserved confounding*) or inadequate measurement or modelling of observed confounders.

In longitudinal studies, it is common to distinguish between *time-varying* and *time-invariant confounding variables*; the former may change value over time for a single individual, and the latter are fixed (or change only in a completely deterministic way, eg, age).^30^

#### Collider bias and selection bias

When two variables both cause a third variable, that third variable is a *collider* (ie, where two arrows ‘collide’ into a third variable on a DAG).^31^ Unlike a confounder, which can cause bias if it is *not* conditioned on, a collider can cause bias if it *is* conditioned on, by opening up a back-door path between the variables entering into it (see figure 1, ‘Collider bias’).

Traditionally in descriptive epidemiology, *selection bias* refers to systematic errors in the process of selecting a representative study sample and has often been thought to primarily affect generalisability of estimates. In causal inference, selection bias more specifically refers to a type of collider bias that occurs when an individual’s presence in the study sample is affected by the exposure and outcome (or variables correlated with these). Since only individuals present in the sample can be included, the study effectively conditions on a collider.^32^

Specific types of collider bias such as ‘Berkson’s bias’, where samples restricted to hospitalised patients can create spurious negative associations between risk factors that are unrelated in the general population, have been recognised for decades.^33^ However, appreciation of the effects of collider bias in general is becoming increasingly important for causal inference.^34^

#### Measurement bias

*Measurement bias* (or *measurement error*) refers to biases that arise because measurements of a variable differ from the (unobserved) true value. *Differential measurement error* arises when the measurement error varies in size depending on another variable and can be represented in a DAG by showing the true (unobserved) value and the measured value as distinct variables.^35^

## Methods for estimating causal effects

### Conventional approaches to confounder adjustment

Causal *effect estimation* refers to quantifying the size of an effect based on available data. The most common causal effect estimation methods in epidemiology typically focus on reducing the impact of confounding by conditioning on some set of common causes of the exposure and outcome. In its simplest form, this can be done by *restricting* the study sample to one level of the confounding variable (eg, only women), *stratifying* (analysing each gender separately) or *matching* (selecting the sample so that the exposed and unexposed groups have the same gender balance). Other methods for confounder adjustment include *multivariable regression* (including confounders as covariates) and *inverse probability of treatment* (or *propensity score*) *weighting.*

### Intermediate confounding and G-methods

*Intermediate confounding* arises when a confounder is affected by prior exposure status.^30^ Conventional methods for confounder adjustment, which hold confounders at a fixed level, are inadequate for handling intermediate confounding for two reasons. First, conditioning on an intermediate confounder blocks part of the effect of prior exposure. Second, conditioning on an intermediate confounder can introduce collider bias, opening additional back-door paths between exposure and outcome.

*G-methods* are a family of methods that address intermediate confounding by taking the observed distribution of intermediate confounders (in the population as well as over time) into account, instead of holding them constant^30 36^; in other words, they estimate marginal effects rather than conditional effects. The following three are G-methods.

*G-computation* (or the *parametric G-formula*) uses a statistical model (eg, a regression model) to predict the potential outcomes (with and without exposure) for each individual observation.^37 38^ This makes it possible to calculate treatment effects in a straightforward way, but relies on the statistical model being correctly specified. *Marginal structural models* aim to make the exposed and unexposed groups exchangeable in terms of confounders by weighting each observation (commonly using *inverse probability of treatment weighting*) so that the distribution of confounders is similar in both groups. An ATE can then be calculated by a simple comparison or unadjusted regression model.^39 40^ *G-estimation* (using *structural nested mean models*) predicts the counterfactual outcome at each time point given no exposure from that point onwards, conditional on prior values of the exposure and confounders.

### Addressing unobserved confounding

The above methods rely on an assumption of no unmeasured confounding (ie, conditional exchangeability), which is often not plausible in observational study designs. The following methods attempt to address unmeasured confounding, subject to certain unprovable assumptions, by exploiting some assignment mechanism (akin to randomisation in an RCT) that determines exposure status but is thought to be unrelated to any unobserved confounders.

#### Instrumental variables (IV)

An IV or *instrument* is a variable that causes some variation in the exposure and is unrelated to the outcome except through the exposure (see figure 1, ‘Instrumental variable’).^41 42^ For example, if a treatment is only performed at certain hospitals, a patient’s distance from such a hospital may affect the probability that they receive this treatment; this distance may then be used as an instrument.^43^ *Mendelian randomisation* uses IV analysis with genetic variants as instruments.^44 45^ IV analysis estimates a local average treatment effect (LATE) among ‘compliers’, that is, individuals whose exposure status is affected by the instrument (table 2). This group cannot be precisely identified, and the LATE may therefore sometimes be of limited practical or policy relevance.^46^

#### Regression discontinuity (RD)

RD methods can be used when exposure status is (wholly or partly) determined by some continuous variable (termed *forcing variable*) exceeding some arbitrary threshold.^47 48^ If the relationship between the forcing variable and the outcome is otherwise continuous, any discontinuity or jump in the relationship can be attributed to the exposure. RD estimates a LATE among the individuals who fall just above or just below the threshold. As with IV analysis, bias can occur if the forcing variable is connected to the outcome through a back-door path or any other pathway besides the exposure.

#### Interrupted time series (ITS)

ITS studies compare the trend over time in a population-level outcome before and after an exposure is introduced.^49^ Assuming that the trend would have been unchanged if the intervention was not introduced, a change in trend at the point of introduction (in terms of level and/or slope) can be attributed to the exposure. ITS can be regarded as a special case of IV or RD, with time being the instrument or forcing variable. ITS addresses time-invariant confounding but can be biased if other events that influence the outcome happen at the same time as the exposure.

#### Difference in differences (DiD)

DiD studies measure the change in a population-level outcome before and after an intervention is introduced, compared with a comparison group where the intervention is never introduced.^50^ This is similar to RD and ITS, but attempts to control for changing time trends, by using a comparison group to represent the counterfactual outcome trend in the exposed. DiD also addresses time-invariant confounding but requires assuming that there would have been no difference in trend between the groups in the absence of the intervention (the ‘parallel trends’ assumption).

## Concluding comments

There is no perfect method for estimating a causal effect in observational data. All methods rely on identifying assumptions, which can sometimes but not always be tested. The practical task is to clearly specify the research question in terms of a causal effect estimand, to choose methods appropriate for this estimand and to carefully interrogate the influence of biases using sensitivity and quantitative bias analysis.

The concepts, methods and formalised principles of causal inference described here are increasingly part of the scientific mainstream. Since questions of causality and causal relationships are fundamental to scientific inquiry, we see this as a welcome shift. However, much of the literature on causal inference methods is highly technical and requires familiarity with concepts from a range of disciplines. Further translational work and resources are needed to make these methods more accessible to and understood by a generalist public health audience.
