# Supplementary material for: Causal inference and effect estimation using observational data
Source: J Epidemiol Community Health. 2022 Sep 6;76(11):960–6. doi: 10.1136/jech-2022-219267 (PMC9554068; doi:10.1136/jech-2022-219267)
Supplement: Supplementary data [file jech-2022-219267supp001.pdf]

Glossary: Causal inference and effect estimation using observational data

Supplemental material

Supplemental Table 1: Examples of common types of potential outcome notation.

Description	Superscript notation[1]	Subscript notation[2]	Functional notation[3]
Potential outcome if exposed (i.e. with exposure A set to 1)[4]	$Y^{a=1}$	Y_1	$Y(1)$
Potential outcome if exposed for an individual *i*	$Y_i^{a=1}$	Y_{1i} or $Y_1(i)$	$Y_i(1)$
Potential outcome if exposed, with mediator M set to 0	$Y^{a=1,m=0}$	Y_{10}	$Y(1,0)$
Potential outcome if exposed, with mediator M set to the value it would have taken if unexposed	$Y^{a=1,M^{a=0}}$	Y_{1M_0}	$Y(1, M(0))$

[1] Hernán MA, Robins JM. Causal inference: what if [Internet]. Boca Raton: Chapman & Hall/CRC; 2020. Available from: https://www.hsph.harvard.edu/miguel-hernan/causal-inference-book/
[2] VanderWeele TJ. A three-way decomposition of a total effect into direct, indirect, and interactive effects. Epidemiology. 2013 Mar;24(2):224–32.
[3] Rubin DB. Causal inference using potential outcomes. Journal of the American Statistical Association. 2005 Mar;100(469):322–31.
[4] Some authors use other symbols than 1 and 0 to denote the possible values of the exposure (e.g. *a* and *a**).
